# Genetic differentiation, local adaptation and phenotypic plasticity in fragmented populations of a rare forest herb

**DOI:** 10.7717/peerj.4929

**Published:** 2018-06-13

**Authors:** Rodolfo Gentili, Aldo Solari, Martin Diekmann, Cecilia Duprè, Gianna Serafina Monti, Stefano Armiraglio, Silvia Assini, Sandra Citterio

**Affiliations:** 1 Department of Earth and Environmental Sciences, University of Milan—Bicocca, Milan, Italy; 2 Department of Economics, Management and Statistics, University of Milan—Bicocca, Milan, Italy; 3 Institute of Ecology, University of Bremen, Bremen, Germany; 4 Museum of Natural Sciences of Brescia, Brescia, Italy; 5 Department of Earth and Environmental Sciences, University of Pavia, Pavia, Italy

**Keywords:** Phenotypic plasticity, Migration, Local adaptation, Evolutionary history, Natural selection, Conservation genetics, Species reinforcement

## Abstract

**Background:**

Due to habitat loss and fragmentation, numerous forest species are subject to severe population decline. Investigating variation in genetic diversity, phenotypic plasticity and local adaptation should be a prerequisite for implementing conservation actions. This study aimed to explore these aspects in ten fragmented populations of *Physospermum cornubiense* in view of translocation measures across its Italian range.

**Methods:**

For each population we collected environmental data on landscape (habitat size, quality and fragmentation) and local conditions (slope, presence of alien species, incidence of the herbivorous insect *Metcalfa pruinosa* and soil parameters). We measured vegetative and reproductive traits in the field and analysed the genetic population structure using ISSR markers (STRUCTURE and AMOVA). We then estimated the neutral (F_ST_) and quantitative (P_ST_) genetic differentiation of populations.

**Results:**

The populations exhibited moderate phenotypic variation. Population size (range: 16–655 individuals), number of flowering adults (range: 3–420 individuals) and inflorescence size (range: 5.0–8.4 cm) were positively related to Mg soil content. Populations’ gene diversity was moderate (Nei-H = 0.071–0.1316); STRUCTURE analysis identified five different clusters and three main geographic groups: upper, lower, and Apennine/Western Po plain. Fragmentation did not have an influence on the local adaptation of populations, which for all measured traits showed P_ST_ < F_ST_, indicating convergent selection.

**Discussion:**

The variation of phenotypic traits across sites was attributed to plastic response rather than local adaptation. Plant translocation from suitable source populations to endangered ones should particularly take into account provenance according to identified genetic clusters and specific soil factors.

## Introduction

In recent years, human-induced habitat loss and fragmentation have caused severe reductions in biodiversity so that many formerly widespread plant species have become rare and are now persisting in isolated populations (Lienert, 2004; Magrach, Larrinaga & Santamaría, 2012; Marcilio-Silva & Marques, 2017). Previous studies highlighted that plants growing in small and fragmented populations may be subject to genetic erosion. This can increase the frequency of (biparental) inbreeding, which can ultimately lead to an inbreeding depression where the genetic load of deleterious recessive alleles reduces individual fitness (Pluess & Stöcklin, 2004; Rosche et al., 2017). However, the long-term effects on population dynamics in perennial herbs can vary when population size is not immediately sensitive to fitness reduction (Tomimatsu & Ohara, 2006).

The reduced link between populations following habitat fragmentation is frequently associated with isolation by distance, isolation by genetic drift, and adaptation to different environments (isolation by environment; see Zhao et al., 2013). Particularly, alteration of environmental conditions accompanying fragmentation (i.e., in abiotic factors such as light regimes, soil conditions, humidity, etc.) may also induce changes in the demographic dynamics of populations (Richards, Emery & McCauley, 2003) and a shift in several phenotypic traits (Jacquemyn et al., 2012). In turn, the shift of phenotypic traits in response to environmental heterogeneity may be driven by genetic (i.e., local adaptation) or non-genetic mechanisms (i.e., phenotypic plasticity). With regard to genetic mechanisms, it is known that some local environmental factors, such as those linked to soil characteristics, can exert a selective pressure on the life history traits of resident populations, causing local adaptation and affecting fitness under the new environmental conditions (Ellis & Weis, 2006); accordingly, neutral genetic differentiation can be explained by environmental factors (Al-Gharaibeh et al., 2016). As regards non-genetic mechanisms, phenotypic plasticity is defined as the ability for an individual genotype to exhibit different phenotypes in response to environmental changes, to which most traits exhibit a plastic response. However, recent models recognise that patterns of plasticity can be either adaptive or non-adaptive with respect to the local phenotypic optimum and such plasticity may consequently influence evolutionary changes in the same traits (Ghalambor et al., 2015).

Individuals from local populations are frequently better adapted to their growth environments than external ones, and their vegetative vigour and reproductive fitness can depend on genetic exchanges present within the population (McKay et al., 2005; Godefroid et al., 2011). Indeed, it is theorised that the success of restoration measures can decrease when the environmental distance increases between the source population and the one needing restoration (Montalvo & Ellstrand, 2000). The source sites where seeds or plant material are collected are generally located within the historical range of target species and have appropriate ecological characteristics with respect to the species’ requirements (Falk, Millar & Olwell, 1996; Possley et al., 2009; Weeks et al., 2011). For these reasons, measuring variation in phenotypic traits, both in terms of reproductive success and vegetative vigour at the population level, as well as investigating the environmental variability of sites in which the populations occur (Pickup et al., 2012; Rúa et al., 2016), are needed to assess the conservation status of a fragmented target species, before implementing translocation measures with the selection of native plant material (Peterson, Hilborn & Hauser, 2014; Diekmann et al., 2015).

Exploring the factors promoting local adaptation in populations is an important part of any restoration attempt as they can provide essential information regarding the complex interactions among habitat selection, dispersal events (i.e., gene flow) and reproductive success (Pickup et al., 2012; Pannell & Fields, 2014; Gibson et al., 2016). It is often assumed that when populations maintain gene flow by migration (i.e., dispersal) and sexual reproduction, they prevent local adaptation; due to homogenising effects of gene flow, the situations under which phenotypic plasticity rather than local adaptation is likely to evolve increase (Torres-Dowdall et al., 2012). For this reason, phenotypic plasticity is also deemed to play a significant role in the population viability of endangered species (Noel, Machon & Porcher, 2007). However, both low fitness of immigrants or habitat loss and fragmentation may reduce gene flow between populations that, in turn, tend to become more adapted to small habitat patches and specific environmental conditions (Garant, Forde & Hendry, 2007).

The restoration of local populations of wide-ranging species and the subsequent selection of potential sources for population reintroduction or reinforcement may be problematic due to the genetic structure and the reduced intra-population genetic variation of such populations (Ellstrand & Elam, 1993; Gentili et al., 2015) that are generally a consequence of the limited dispersal ability of the species (Maschinski & Haskins, 2012; Orsenigo et al., 2017). In this framework, conserving the species’ genetic diversity is fundamental, as it is associated with its evolutionary potential and viability as well as with its ability to adapt to local and global environmental changes (Reed & Frankham, 2003). Moreover, introducing or transplanting genotypes with lower fitness than local genotypes can have negative consequences for the establishment success of restored populations (Hufford & Mazer, 2003). In such cases, pre-reintroduction genetic analysis is considered good practice in ecological restoration (Godefroid et al., 2011).

The quantification of population differentiation based on neutral genetic markers and quantitative traits can highlight the relative role of evolutionary processes such as natural selection, genetic drift and gene flow for patterns of local adaptation (Brommer, 2011; Leinonen et al., 2013). Fixation index (F_ST_) is widely used to estimate genetic differentiation with neutral loci (SSR, ISSR, AFLP) by analysing variance in allele frequency (Wright, 1949). In contrast, phenotypic differentiation index (P_ST_) is an estimate of quantitative genetic differentiation (i.e., additive genetic variance) using quantitative trait measurements within populations (e.g., plant size, growth rate, etc.; Brommer, 2011). The P_ST_ index assesses local adaptation through natural selection of wild populations and is an approximation of the quantitative genetic differentiation index (Q_ST_), obtained in common garden experiments, but influenced by environment (Brommer, 2011). The relationship between the values of P_ST_ and F_ST_ can be used to estimate the relative importance of genetic processes and selection: (a) P_ST_ = F_ST_ indicates that divergence is compatible with a scenario of genetic drift; (b) P_ST_ > F_ST_ indicates directional selection (i.e., when one extreme phenotype is favoured over other ones) among populations; (c) P_ST_ < F_ST_ indicates that the same phenotypes are favoured in different populations due to stabilising selection.

In this study, we explored phenotypic plasticity, the divergence in quantitative traits (i.e., local adaptation) and the patterns of genetic variation at the population level of the species *Physospermum cornubiense* (L.) DC. a herb, which is rare due to habitat loss and deterioration in the fragmented woodlands of the Po plain (N-Italy). Possible restoration actions (reintroduction or translocation) for *P. cornubiense* will need to consider a number of viable populations as propagule donors within the species’ historical range to (re)create new or reinforced populations to increase the range and reduce the risk of extinction.

In order to inform restoration efforts for *P. cornubiense*, we quantified the genetic and phenotypic variation of populations in the Italian portion of the species’ range. We hypothesised that historical habitat loss and fragmentation have influenced genetic structure and local adaptation. Additionally, the long-term survival of some populations may be threatened by the loss of genetic diversity and declines in reproductive fitness. To address these questions, we used complementary approaches: (a) we measured a set of morphological traits to assess phenotypic population differentiation in the field, correlating local and landscape conditions with the population characteristics; (b) we investigated genetic diversity and genetic differentiation among populations by examining F_ST_ values; (c) we assessed the patterns of local adaptation, in terms of level of divergence of quantitative traits, by applying a P_ST_ approach.

## Material and Methods

### Study species and area

*Physospermum cornubiense* (L.) DC. is a rhizomatous perennial (hemicryptophyte) herb, belonging to the Apiaceae, in which it represents a relatively isolated taxon compared with other genera of the family (Downie, Katz-Downie & Watson, 2000). It reproduces by outcrossing in addition to a small amount of vegetative spread (Online Atlas of the British and Irish flora: http://www.brc.ac.uk/plantatlas/). Its chromosome number (2*n*) ranges from 2*n* = 14 to 2*n* = 22 (Löve, 1978; Vasilyeva, Retina & Pimenov, 1981). During this study, flowers of *P. cornubiense* were observed to be visited by several groups of insects (belonging to Diptera, Cerambicidae and Lepidoptera), which are well known to disperse pollen over medium to long distances (Ghazoul, 2005) allowing a rapid pollen transfer. The species is a European temperate element with a discontinuous distribution across different biogeographic regions, ranging from the Black sea and the Balkan peninsula to Italy, the north-western Iberian peninsula and the southern United Kingdom (Oberdorfer & Hofmann, 1967). In the Po plain region (Italy), *P. cornubiense* populations are peripheral and highly fragmented due to historical habitat loss and deterioration. Although declining, the species is not included in protection programmes (Regione Lombardia, 2010).

**Figure 1:**
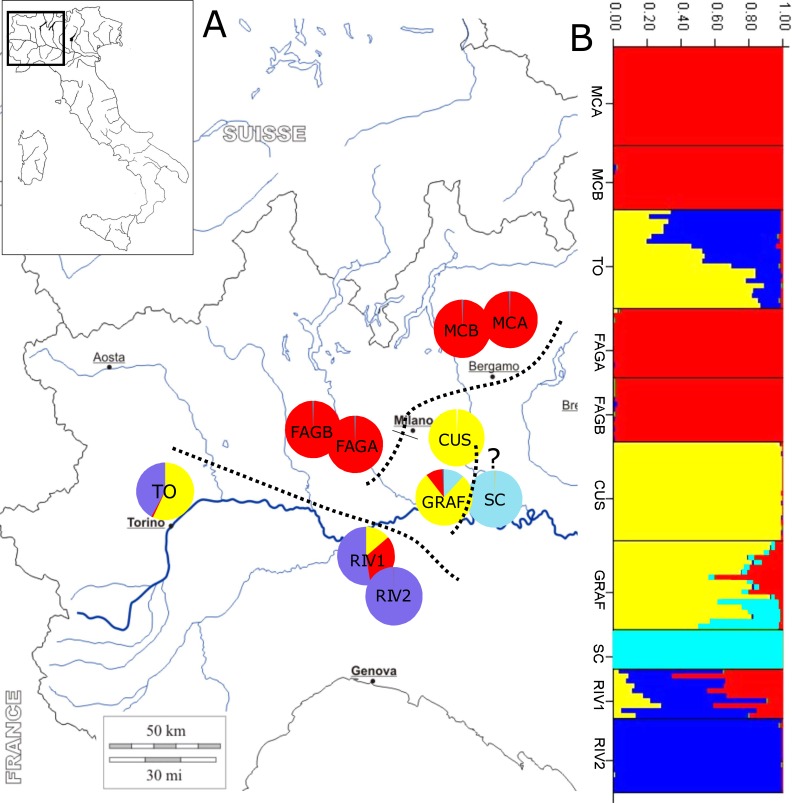
Location of sample sites and genetic clusters. (A) Geographic location of populations and STRUCTURE analysis; dotted lines subdivide the geographic populations resulting from the analysis: upper Po plain (MCA, MCB, FAGA, and FAGB), lower Po plain (CUS, GRAF, and probably SC, labelled with a question mark), and Apennine/Western Po plain (RIV1, RIV2, and TO). (B) Bar diagram representing the proportion of ancestry in each of the K populations.

The study area falls in the Po plain (Northern Italy; Fig. 1A) in a territory of about 9,000 km^2^, from western Piedmont to Lombardy regions (45°06′N; 08°49′E). Here, *P. cornubinese* populations were found to grow in shaded or semi-shaded conditions and are mainly found in oaks (*Quercus petraea* (Matt.) Liebl. and *Q. robur* L.) or chestnut woods (*Castanea sativa* Miller) mixed forest, preferring moderately moist soils with low pH (Preston, Pearman & Dines, 2002).

**Table 1:** Main environmental characteristics of 10 populations of Physospermum cornubiense. The whole dataset of environmental variables is reported in Table S1.

Code	Locality	Habitat	Habitat size (ha)	Elevation (m)	Slope (°)	pH	Mg (mg/ 100 g soil)	Ca (mg/ 100 g soil)	Tot N%	Population size (*n*; estimate)	Flad%	Non_Flad%	Seedl/juv%
GRAF	Graffignana	Mixed oaks; chestnut	4	130	30	3.6	8.7	17.3	0.090	50	44.0	30.0	26.0
SC	San Colombano	Chestnut	1	160	15	3.5	7.0	23.9	0.160	16	18.8	62.5	18.8
RIV1	Rivanazzano (N)	Mixed oaks	2	235	20	4.2	16.2	84.7	0.155	32	40.6	18.8	40.6
RIV2	Rivanazzano (SW)	Mixed oaks	4	370	25	4.1	30.2	69.6	0.113	378	24.9	48.1	27.0
FAGA	Fagiana A	Mixed oaks	3	126	0	3.7	4.3	18.3	0.341	236	38.1	30.9	30.9
FAGB	Fagiana B	Mixed oaks	2	111	0	3.6	3.1	11.1	0.503	67	11.9	46.3	41.8
MCA	M.te Canto	Chestnut	7,5	330	27	3	14.4	92.8	0.230	400	16.0	40.8	43.3
MCB	M.te Zocca	Mixed oaks	5	470	20	4.2	15.8	130.1	0.283	84	26.2	40.5	33.3
CUS	Cusago	Mixed oaks	12	140	0	2.4	5.6	23.9	0.399	209	4.3	69.9	25.8
TO	Collina di Superga	Chestnut	4	300	30	4.1	43.6	25.0	0.186	655	64.1	29.0	6.9

**Notes.**

Abbreviations Flad Proportion of flowering adults% Non_Flad% Proportion of notflowering adults seedl/juv% Proportion of seedlings/juveniles

### Quantitative traits data

The data were collected in ten natural populations of *P. cornubinese* across the study area (Table 1). To compare the relative performance of populations, we collected data on abundance and traits from at least 15 adult individuals in each population, where possible. Indeed, in populations with small population size and for individuals highly damaged by insects, collecting information on traits was not possible (i.e., population with very small population size). For each population, data were collected from plants intercepted along 15 transect lines (of different length due to the irregular shape of the sampling area), each selected with a random starting point and a randomly selected direction, covering the whole population range (see Burnham, Anderson & Laake, 1980).

The data collection of the different traits was carried out during the different phases of the vegetative season, particularly in the months of July (end), August and September (beginning) 2013. The data collection included:

(a) *abundance*: counting of individuals, total for the population (Pop_sz) and per age class including seedling, juvenile, non-flowering adult and flowering adult. Because of the difficulty of distinguishing seedling and juvenile stages, these two classes were grouped in the analyses.
(b) *vegetative traits*: plant height (cm; Pl_H), measured from the ground to the highest growing point of the main branch; lateral spread (cm; Lat_spr), measured as the maximum diameter of the plant; number of leaves (N_leav).
(c) *reproductive traits*: maximum size of a composite umbel (cm; Infl_sz); total number of composite umbels (Comp_umb); total number of simple umbels (Simp_umb); seed mass (g; SW), measured with a precision balance (0.0001 g), based on ten randomly collected seeds per individual.

### Environmental variables

To investigate the contribution of environmental factors to the differentiation of *P. cornubiense* populations, we collected data on landscape variables (LAND) and local variables (LOC) (Table S1). The LAND variables were: (a) habitat type (class; mixed oak woodland = 1, chestnut woodland = 2); (b) habitat size (ha); (c) habitat quality (class: low = 1, medium = 2, high = 3)–an expert-based scale related to the presence and abundance of herb species typical of habitats with *P. cornubinese*; (d) habitat fragmentation index (class: low = 1, medium = 2, high = 3)–this index is based on the distance between populations, perforation (formation of holes in habitat patches), dissection (presence of narrow linear corridors) and shrinkage (information on habitat shape); see Table S1 for the scoring procedure). The LOC variables were (a) elevation (m.a.s.l.); (b) slope (°); (c) presence of alien plant species (class: *y* = 1, *n* = 0); (d) presence of the herbivorous *Metcalfa pruinosa* Say, 1830, an invasive polyphagous insect (Homoptera) native to North America that colonised Europe from 1979 onwards (Strauss, 2010; class: *y* = 1, *n* = 0); (e) soil characteristics (pH, the concentrations of Ca, K, Mg, P, C and N, the C/N ratio).

Regarding soil characteristics, soil samples were collected and analysed applying standard techniques (according to the protocol of Wehling & Diekmann, 2008). Soil samples were collected in 2013. In each patch of a population, five 4 cm deep soil cores were collected from the upper soil below the litter layer and pooled to one sample. Each sample was air-dried to constant mass and sent to the soil laboratory of the Institute of Ecology at the University of Bremen. Here, the soil from all samples was passed through a 2 mm sieve for further analysis. For the determination of pH, 10 g of soil were mixed with 25 ml of 0.01 M CaCl_2_ and the solution was placed on a shaker for 2 h, before measuring pH with a standard glass electrode. The total contents of carbon (C) and nitrogen (N) were determined in % using an elemental analyser (Euro EA, Hekatech, Germany; instrument’s software: Callidus). The cations calcium (Ca), potassium (K) and magnesium (Mg) as well as plant available phosphorus (P) were extracted with ammonium lactate. The concentrations of the three cations were measured with Atomic Absorption Spectroscopy (AAS, PU9100, Philips, Holland; instrument’s software: PU9100 Analytics), whereas *P* was determined by flow injection analysis (FIA, Star5000, Tecator, Sweden; instrument’s software: SuperFlowDue v. 1.4). The laboratory measurements followed the methods described by Suchopar (2014). The soil variables used in the statistical analysis included the C/N ratio.

### Sampling material and DNA extraction

Within each population, 8–20 individuals were sampled depending on the population size. In total we sampled 151 individuals. For each population, data were collected from plants intercepted along 20 transect lines (of different length due to the irregular shape of the sampling area), each selected with a random starting point and a randomly selected direction, covering the whole population range. Table 1 shows the localities and the main characteristics of the sampled populations.

All sampled individuals were established adults; plant material (leaves) was collected at a reciprocal distance of at least 10 m. Genomic DNA was extracted from the dried leaves using the EuroGold Plant DNA Mini Kit (Euroclone, Pero, Italy) following the manufacturer’s instructions. The quantity and quality of the extracted DNA were determined by absorbance measurements (nanophotometer NP80, Implen, Munich, Germany).

### ISSR markers

We used ten inter-simple sequence repeat (ISSR) molecular markers based on a technique involving the use of microsatellite sequences as primers in a PCR analysis to generate dominant multilocus markers. ISSR markers combine the advantages of AFLP and microsatellites (SSRs): they are highly polymorphic as they use universal primers and are useful for organisms for which genetic information is scarce (Pradeep Reddy, Sarla & Siddiq, 2002). During the scoring, bands/peaks are recorded into the binary symbols, with “1” for band/peak presence and “0” for band/peak absence. The ISSR primers used in this study were assessed from an initial pool of 20 universal primers. Among these, eight were selected according to their reproducibility and degree of polymorphism: UBC807, UBC808, UBC811, UBC825, UBC834 UBC841, UBC842, UBC847 (see Table S2 for primer sequences). The Polymerase Chain Reaction (PCR) was performed in a 12.5-µl total reaction volume, including 15 ng total genomic DNA, 1.5 µL of 10X reaction buffer (Qiagen, Germany) and 5 µM of 17/18 bp primers, 0.15 mM dNTPs in equal ratio and one unit (per reaction) of TopTaq DNA-polymerase (Qiagen, Germany). The ISSR primers were fluorescently labelled with 6-FAM (6-carboxy fluorescein) at the 5′ terminal. The PCR was performed (in a Mastercycler Gradient thermal cycler) under the following temperature profile: 94 °C for 5 min. for the initial denaturation, followed by 40 cycles of 35 s. at 94 °C, for 45 s. at 54 °C, 90 s. at 72 °C and finally, one cycle at 72 °C for eight min.

The ISSR products were first separated by electrophoresis in TBE buffer and visualised using 1.5% agarose gel (Roche, Berlin, Germany). Then, aliquots of 2 µL of ISSR PCR products were suspended in 9.75 µL of deionised formamide and with 0.25 µL of size standard (Genescan^®^, LIZ1200, Applied Biosystems, Foster City, CA, USA) for capillary electrophoresis using an ABI PRISM 3100 Genetic Analyzer (Applied Biosystems, Foster City, CA, USA). Analysis of the results was performed using the software Gene Mapper 4.0 and Raw Geno 2.0 (an R package for automating fragment analysis; see Arrigo et al., 2009), checking for possible differences or incongruences in the peak detection, following the specific instructions for ISSR raw fluorescent data (Applied Biosystems Application Note, 2010). The automated scoring identified 186 peaks of which only 171 were retained for further analysis after the quality check.

### Genetic analyses

Common genetic diversity measures at the population level were calculated using POPGENE v. 1.31 such as: percentage of polymorphic loci (%P), Nei’s genetic diversity (H_Nei; Nei, 1973; Nei, 1978; H), and gene flow (Nm) (Yeh et al., 1997). The presence/absence matrix generated after ISSR analysis was subjected to a neighbour-joining (NJ) analysis based on a Nei-Li distance matrix that was created with the software TREECON v. 1.3b (Van de Peer & De Wachter, 1994). The NJ tree was graphically edited using the SplitsTree v. 4.13 software (Huson & Bryant, 2006). The significance of nodes was calculated with 1,000 bootstrap replicates.

The population structure of *P. cornubiense* at the landscape level was inferred and individuals assigned to geographical ancestry populations by using the software STRUCTURE v. 2.3.4., which provides the use of dominant markers such as ISSRs (Pritchard, Stephens & Donnelly, 2000; Falush, Stephens & Pritchard, 2007). The allele frequencies of the different *P. cornubiense* populations were assumed to be correlated, which is a realistic assumption for populations that are likely to be similar due to common migration events or shared ancestry. STRUCTURE results were set on an admixture model where individuals may have mixed ancestry. To determine the optimal number of clusters, 20 independent runs for each value of *K* (*K* = 1 to 10) were performed with an admixture model at 100,000 runs of burnin period and 500,000 Markov chain Monte Carlo iterations. Before the end of the burnin period, we checked for F_ST_ and alpha plots to be sure that the summary statistics had a stable trend. To determine the number of clusters we used Δ*K*, the second-order rate of change in lnP(*X*—*K*), for successive values of *K* (Evanno, Regnaut & Goudet, 2005).

The genetic structure of populations was also investigated, estimating F_ST_ according to Wright’s F-statistics, with AFLP-SURV v.1.0 (Vekemans, 2002). This analysis was performed following a Bayesian approach (non-uniform prior distribution of allele frequencies) and assuming Hardy–Weinberg equilibrium. Statistical significance was determined using 10,000 permutations.

Analysis of molecular variance (AMOVA) was carried out using the Genalex v. 6.5 (Peakall & Smouse, 2005) in order to estimate the genetic structure and genetic differentiation within populations and among populations. The significance of the estimates was calculated through 9,99 random permutations. AMOVA allowed the calculation of the Φ_ST_ values derived with Genalex, being the Φ_ST_ value an estimation of F_ST_ for dominant data (Peakall & Smouse, 2005).

### Phenotypic and genetic differentiation

To estimate the role of local adaptation through natural selection in the *P. cornubiense* populations, comparisons of quantitative traits and genetic differentiation among populations were performed. According to Leinonen et al. (2006) and Brommer (2011), when Q_ST_ estimates are not available, P_ST_ can be justified as a substitute. According to Brommer (2011) “*divergence across populations of species that are less amenable for proper* Q_ST_ *estimation may still be of considerable evolutionary or conservation interest*” and it can be assessed by using P_ST_. In our study, we chose P_ST_ index since the seeds of *P. cornubiense* were difficult to germinate and some populations had a very small number of flowering individuals and consequently did not produce an adequate number of seeds that could be used for common garden experiments.

For each population pair, pairwise P_ST_ values were calculated for each trait (and for an average P_ST_), using the following formula: PST = *cσ*^2^_B_∕(*cσ*^2^_B_ + 2*h*^2^*σ*^2^_W_). In this formula, *σ*^2^_B_ and ${\sigma }_{\mathrm{W}}^{2}$ are the between-population and within-population variance components for a trait, respectively; *h*^2^ expresses the heritability (the proportion of phenotypic variance that is due to additive genetic effects); the scalar c expresses the proportion of the total variance that is presumed to be due to additive genetic variance across populations (Brommer, 2011; Leinonen et al., 2013). The problems of using P_ST_ as an approximation of Q_ST_ are well known in the literature and mainly caused by the difficulty of performing an accurate estimation of the parameters *c* (proportion of the total variance) and *h*^2^ (heritability) in the wild, for a set of traits (Pujol et al., 2008). Consequently, Brommer (2011) recommended assessing the strength of P_ST_–F_ST_ comparisons exploring the variation range of c and *h*^2^ values, where *c* ≤ *h*^2^ and ( 0 < *c*∕*h*^2^ ≤ 1). Therefore, a sensitivity analysis of the P_ST_–F_ST_ comparisons, starting from the null assumption of *c* = *h*^2^, was carried out, accounting for the lower limit of the 95% CI of F_ST_.

### Statistical analysis

The difference in the frequency distribution of the age classes across populations was tested using *χ*^2^ statistics (contingency table). To analyse the correlation between the environmental factors (LAND and LOC, including soil characteristics) with vegetative and reproductive traits of *P. cornubiense* populations, a generalised linear mixed model (glmm) with a normal or Poisson or binomial distribution (depending on the variable) was carried out. To analyse the correlation between LAND and LOC and demographic and genetic variables of populations (constant variables), a generalised linear model (glm) with Gaussian distribution was carried out. We performed corrections for multiple tests by using the Holm’s method (Holm, 1979) for family-wise error control, separately for each family of tests (family A included demographic and genetic [see following sub-section] variables and family B included species traits variables; see Table S3).

The relationship between measures of reproductive success (Simp_umb, Comp_umb, Infl_sz and SW) and Nei’s genetic distance (H_Nei) with demographic parameters (FLad, Pop_sz, etc.) were also investigated by performing Pearson’s correlation analysis, correcting for multiple testing.

LAND and LOC matrices were then merged to create an overall environmental matrix (ENV); all three matrices were retained to perform successive separate tests. To assess whether levels of quantitative genetic differentiation in wild populations (P_ST_), genetic differentiation (F_ST_), geographic distances (GEO) and environmental (LAND, LOC and ENV) variability were related, we calculated pairwise correlations between the correspondent distance matrices, applying Mantel tests (Mantel, 1967) with 9,999 permutations in ARLEQUIN version 3.5 (Excoffier & Lischer, 2010). Environmental distance matrices were generated from LAND, LOC and ENV calculating Euclidean distance between all the population pairs. Mantel tests for quantitative genetic components (P_ST_) were also carried out. We then performed corrections for multiple tests by using the Holm’s method (Holm, 1979) for familywise error control, separately for each family of tests (family A included only trait variables from P_ST_ and F_ST_ matrices and family B included P_ST_ and F_ST_ matrices versus GEO, LAND, LOC and ENV matrices; see Table S3).

If not otherwise specified, statistical analyses were performed in R 3.0.3 (R Development Core Team, 2015), using the pakages multcomp, hmisc and lme4.

## Results

### Population characteristics

The *P. cornubiense* populations occurred in forest patches varying in size from 1 ha to 12 ha. Soil pH ranged from 3.5 to 4.2. Mg and Ca contents greatly differed between the sites, ranging from 3.1 mg 100 g^−1^ to 43.6 mg 100 g^−1^ and from 11.1 mg 100 g^−1^ to 130.1 mg 100 g^−1^ respectively, and the values of the two elements were positively correlated with each other (Spearman’s rho = 0.68; *p* = 0.028). Mg and Ca contents were also positively correlated with pH (Spearman’s rho = 0.745; *p* = 0.013; Spearman’s rho = 0.733; *p* = 0.015). Total N was very low in the GRAF population but relatively high in FAGB, with proportions of 0.09% and 0.50%, respectively (Table 1). The full results of the soil analyses (including correlations between variables) are provided in the Table S1.

As regards to demographic data, the number of individuals in the populations ranged from 16 in SC to 655 in TO. The proportions of individuals of different age classes of *P. cornubiense* clearly differed among sites (Table 1; *χ*^2^ = 299.01; *df* = 18; *p* < 0.001). In particular, the percentage of seedlings/juvenile plants was lowest in TO (6.9%) and highest in MCA (43.3%); the proportion of flowering adults was lowest in CUS (4.3%) and highest in TO (64.1%).

**Figure 2:**
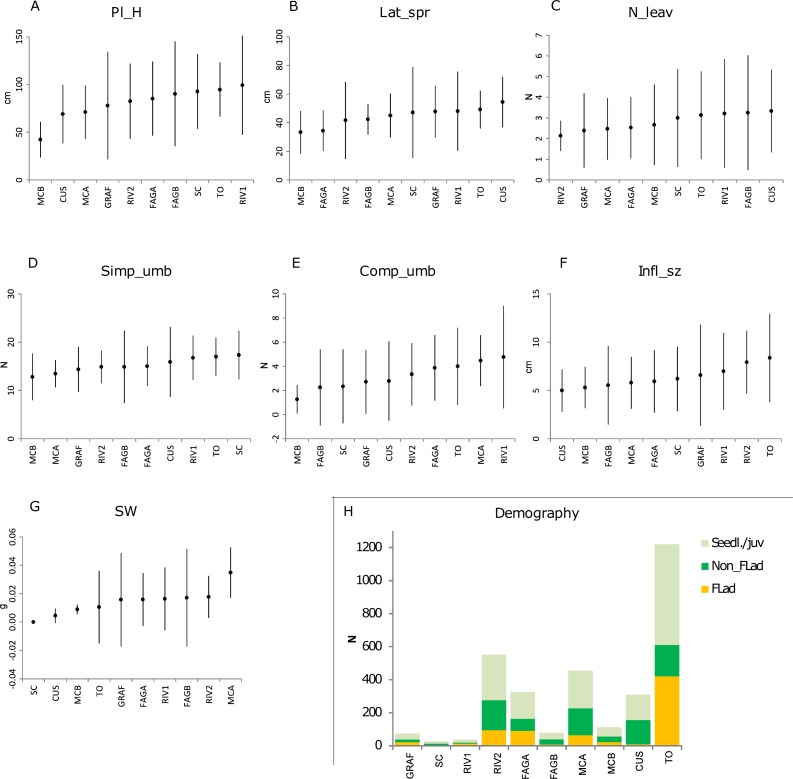
Phenotypic traits and demography of *P. cornubiense* populations. (A) Reproductive and vegetative traits within each population, ordered in increasing order of the magnitude of their estimated mean (mean ± 2*sd); abbreviation: Pl_H, plant height (A); Lat_spr, lateral spread (B); N_leav, number of leaves (C); Simp_umb, total number of simple umbels (D); Comp_umb, total number of composite umbels (E); Infl_sz, maximum size of a composite umbel (F); SW, seed mass (G). Age class distributions of populations (H); Abbreviation: Seedl./juv., seedling/juvenile; Non_flad, non-flowering adult; Flad, flowering adult. Note*: In SC population the measure of reproductive traits are the result of a small number of individuals (*n* = 3).

The differences between populations, with respect to their vegetative vigour and reproductive fitness components, are shown in Figs. 2A–2G. In general, regarding vegetative components (plant height, lateral spread and number of leaves), the highest values were found in TO and CUS, while the lowest values were encountered in MCB. For instance, lateral spread was highest in CUS and TO (54.4 and 49.2 cm respectively) and lowest in MCB (33.3 cm). A less evident pattern was found for the reproductive fitness components (maximum size of a composite umbel, total number of simple umbels, inflorescence size and seed mass). As regards to the SW trait, no seeds were found in SC, while the small population at MCB exhibited a low value (0.0089 g). This pattern was also reflected in the seedling recruitment of populations (Table 1; Fig. 2H).

### Correlation of environmental factors with population traits

The glmm and glm, after corrections for multiple tests by using the Holm’s method, revealed that soil factors and habitat characteristics were correlated with population abundance and traits (Table 2). In particular, glm highlighted that Mg concentration was positively correlated with the number of flowering adults (FLad: *G* = 18.204; *p* < 0.001) and population size (Pop_sz: *G* = 12.053; *p* < 0.001).

**Table 2:** Summary of statistics from glm and glmm relating LOC/LAND variables to population demography/species traits. Only significant relationships are reported; full list of correlations, including glmm, is reported in Table S3. Numbers in parentheses are adjusted *p*-values according to Holm’s correction for multiple comparisons.

Dependent variable	Explanatory variable	Slope	Intercept	*G*	*df*	*p*-value (corrected value)
Pop_sz	Mg	12.582	25.326	12.053	1	0.0005 (0.04029)
FLad	Mg	8.153	−46.821	18.204	1	0.0001 (0.015)

No significant relationships were detected by glmm analyses.

### ISSR genetic analyses and population structure

The ISSR analysis performed on the ten *P. cornubiense* populations produced a total of 171 scorable peaks from about 50 bp to 1,000 bp. The percentage of polymorphic loci ranged from 22.81% in MCB to 61.99% in GRAF while Nei’s genetic diversity varied from 0.071 in MCB to 0.1316 in CUS; the mean H Nei at the species level was 0.109 (Table 3). Gene flow was Nm = 0.94.

**Table 3:** Genetic diversity of 10 populations of *Physospermum cornubiense*. The standard deviation is shown in parenthesis.

Code	Locality	Sampled individuals (*n*)	% *P*	H Nei	UH Nei	No. private bands
GRAF	Graffignana	18	61.99%	0.130	0.134	7
SC	San Colombano	8	28.07%	0.087	0.092	0
RIV1	Rivanazzano (N)	10	38.60%	0.114	0.120	2
RIV2	Rivanazzano (SW)	15	44.44%	0.119	0.123	3
FAGA	Fagiana A	14	32.75%	0.099	0.103	1
FAGB	Fagiana B	13	42.11%	0.121	0.126	2
MCA	M.te Canto	20	29.24%	0.084	0.086	3
MCB	M.te Zocca	13	22.81%	0.071	0.074	1
CUS	Cusago	20	61.40%	0.155	0.159	12
TO	Collina di Superga	20	39.77%	0.112	0.115	0
Mean		15,1 (±4.3)	40.12% (±13.2)	0.109 (±0.025)	0.113 (±0.025)	3.1 (±3.7)

**Notes.**

Abbreviations *P*% Proportion of polymorphic loci H Nei Nei’s genetic diversity UH Nei Unbiased Nei’s sgenetic diversity

Overall, no significant correlations were found between genetic diversity and population size, as well as between genetic diversity and reproductive fitness, even if some of the smallest populations (SC and MCB) showed both low genetic diversity and/or reproductive fitness. The relationship among the *P. cornubiense* populations and individuals was also investigated by a NJ analysis (based on the Nei-Li distance) and performed at the individual level (File S1).

STRUCTURE analysis estimated the highest mean log likelihood at *K* = 5 [ln*P*(*D*)(−10, 971.062)], indicating that populations of *P. cornubiense* are subdivided into four different genetic geographic groups (Fig. 1; File S1). A certain degree of structure in *P. cornubiense* populations and a subdivision in three main geographic groups, plus the divergent cluster of the small SC population, is evident: (a) upper Po plain (FAGA, FAGB, MCA, MCB); (b) lower Po plain (CUS, GRAF and the divergent SC); and (c) Apennine/Western Po plain (RIV1, RIV2, TO).

The overall genetic differentiation among populations according to Wright’s *F*-statistics was F_ST_ = 0.365. Pairwise F_ST_ ranged from 0.101 (FAGA vs. FAGB) to 0.628 (MCB vs. SC). AMOVA analysis showed that 66.1% (estimated variance = 0.409; *p* < 0.001) of the total genetic variation is attributed to individuals within populations, while 31.2% (estimated variance = 4.785; *p* < 0.001) and 2.7% (estimated variance = 10.124; *p* < 0.001) are due to differences among populations and between regions, respectively. The Φ_ST_ value estimated with AMOVA was 0.339.

The Mantel test between the neutral genetic differentiation (F_ST_) and geographic distance did not give a significant result following corrections for multiple tests by using the Holm’s method (see Table S1 and Table S4).

#### PST-FST comparisons and correlation with environmental factors

**Figure 3:**
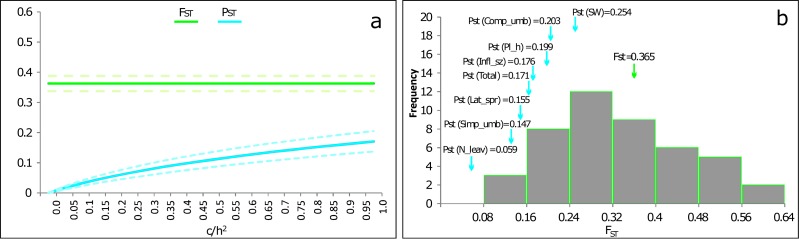
PST values of phenotypic traits of *P. cornubiense* as a function of their genetic differentiation among populations. (A) Overall value PST (with confidence intervals of 95%) combining quantitative traits of *P. cornubiense* as a function of the c/h2 ratio where c represents the proportion of the total variance and h the heritability. The lowest value of c/h2 for which PST exceeds FST (i.e., critical value of *c*∕*h*^2^ according to Brommer, 2011) can be considered an indication of the robustness of using PST as an alternative for QST. In the figure, the FST value (green lines; dotted lines represent the confidence intervals of 95%) exceeds PST (blue lines; dotted lines represent the confidence interval of 95%) for all *c*∕*h*^2^ values, indicating no evidence of local adaptation. (B) The different values of PST (blue arrows) for different traits along the FST (green arrow) distribution for neutral loci.

The P_ST(Total)_ was 0.171 (95% CI [0.137–0.205]). This value was significantly lower than F_ST_ (ANOVA: *F*_1,88_ = 40.57; *p* < 0.001) as also shown in the P_ST_–F_ST_ comparison function of *c*∕*h*^2^ (Fig. 3). None of the P_ST_ results across the measured traits exhibited a value higher than F_ST_ (Fig. 3B).

Applying a Mantel test to F_ST_, P_ST_ andgeographic or environmental distance matrices (Table S4) we investigated possible patterns between all population pairs (*n* = 10) of *P. cornubiense*. No patterns were found.

## Discussion

Our results showed that historical habitat loss and deterioration across the range of *P. cornubiense* in the Po plain region influenced the genetic structure of the populations but not their local adaptation. Therefore, differences in the phenotypic traits across sites could be due to a plastic response of populations to local environmental conditions. Despite no overall correlation was found between genetic diversity and population size and between genetic diversity and reproductive fitness, two among the smallest populations (SC and MB) showed low genetic diversity and low reproductive fitness; therefore, their long-term survival is compromised and may require restoration measures.

### Environmental gradients vs *P. cornubiense* populations

Soil characteristics had an influence in shaping the demographic patterns and the traits of the studied populations. Populations of *P. cornubiense* exhibited morphological variation in response to Mg content in the soil*.* Increasing Mg content was positively correlated with the number of flowering adults and with population size. Verbruggen & Hermans (2013) highlighted the key role of Mg availability in several plant functions; a lack of Mg negatively influences photosynthetic efficiency, plant growth and productivity. As demonstrated in previous studies, soil properties modulate the ability of the individuals in a population to grow and persist according to their specific tolerances and preferences with respect to soil components (Smith et al., 2012; Schweitzer et al., 2014). At the community level, differences in soil characteristics may shift the competitive balance between plants and/or soil microorganisms (Rennenberg et al., 2009). In any case, some confounding factors should be considered when interpreting these results, such as: a) pH variation (in our results positively correlated with Mg content), which could influence the availability and uptake of Mg and other key elements (Dighton & Krumins, 2014); and b) the complex interactions between biotic and abiotic factors that occur within the soil ecosystem.

### Genetic diversity and structure

Intra-population genetic variation was moderate, as several populations of the studied species exhibited Nei’s genetic diversity values lower than 0.1. Such levels of genetic diversity seem to be in accordance with the trend observed for species with fragmented populations or with a restricted range (Frankham, 1997; Frankham, 1998). In addition, this reduced genetic variability within populations has already been observed in other perennial outcrossing species with a preference for sexual reproduction (Nybom, 2004). On the other hand, such levels of genetic diversity seem to be lower than those observed in other species belonging to the Apiaceae when using dominant markers such as in *Petagnaea gussonei* (Spreng.) Rauschert (mean H Nei = 0.136; (De Castro et al., 2013), *Changium smyrnioides* Wolff (diversity value = 0.488; Fu, Qiu & Kong, 2003) and *Eryngium alpinum* L. (mean H Nei = 0.199; Gaudeul et al., 2004).

*P. cornubiense* exhibits evident geographic structure, reflecting the historical fragmentation of the species range across the biogeographic province of the Po plain, and highlighting isolation, reduced gene flow and genetic drift among populations. The resulting genetic clusters of *P. cornubiense* can be assigned to the upper Po plain, lower Po plain (including the SC divergent cluster), and Apennine/Western Po plain and may best be explained by historical genetic drift. The very small and endangered SC population likely originated from the same cluster of CUS and GRAF but it is probably a sister population to GRAF (i.e., close populations with common origin). The SC population has probably been exposed to reduction and deterioration of habitat (low values of habitat quality and habitat size) causing separation between adjacent forest patches. Subsequently, the population has been subject to isolation within a smaller patch and with a reduction in population size and genetic divergence without clear evidence of an inbreeding depression. However, we found some low values of H Nei in the smallest population (SC), which also did not produce any inflorescences and consequently, seeds. The origin of such genetic clusters is probably a consequence of isolation due to habitat loss and fragmentation combined with population differentiation, indicating scarce gene flow. Processes of isolation by distance occur when gene flow and gene drift are in equilibrium and when gene flow decreases with distance among locations (Hutchison & Templeton, 1999). In any case, increased longevity (*P. cornubiense* is a perennial species) may have reduced the effects of genetic erosion. Indeed, under unstable population sizes, perennial species may exhibit low levels of biparental inbreeding and reduced drift due to their longevity and overlapping generations (Rosche et al., 2016).

It is well known that in historical pre-Roman times, the Po plain was covered by a mixed oak-hornbeam-elm forest that has now been almost entirely destroyed after thousands of years of land exploitation as a result of urbanisation and agriculture (Ruffo, 2001). In the past, the *P. cornubiense* populations were closely interconnected with each other: not isolated or fragmented. In the literature, the reduced genetic variability of small fragmented populations (like SC in our study) with high inbreeding and genetic drift is recognised that it depresses population fitness and viability in recently diverged populations (Gentili et al., 2010; Neaves et al., 2015).

The poor connectivity among habitats and the apparent lack of mechanisms for long-distance seed dispersal may have restricted genetic exchange among populations. According to Honnay et al. (2005) the limited dispersal between fragments along with long generation times, prolonged clonal growth and limited seedling recruitment, may interact in determining population genetic differentiation that can reach a degree of 30–40% in perennial forest herb species. These same factors may also have acted in determining the population structures of the studied species, favouring genetic divergence by isolation.

### PST-FST comparisons and local adaptation

Overall, the genetic population differentiation in our study was pronounced ( F_ST_ = 0.365) supporting genetic divergence by isolation (Hornemann, Michalski & Durka, 2012). According to Charlesworth (1998), high F_ST_ values can also be seen as an expression of the low diversity within populations, as we observed in our study. Regarding the phenotypic variation via genetic mechanisms, there was scarce evidence of local adaptation in vegetative and reproductive traits among the fragmented populations. Indeed, the fact that Pst <Fst for all of the comparisons indicates that these are subject to stabilising selection, which has maintained approximately the same phenotypic characters across different habitats/landscapes despite the likely presence of genetic divergence by isolation (Brommer, 2011; Leinonen et al., 2013). This lack of phenotypic differentiation via genetic mechanisms may have different causes: (a) it may indicate that stabilising selection, in which either the same alleles or the same genotypes have been favoured in different populations experiencing similar overall environmental conditions that have acted over a long period (Loveless & Hamrick, 1984); or (b) it may reflect historical patterns of population structure (Musche, Settele & Durka, 2008) and recent fragmentation.

When both genetic and environmental distances increase with geographic distance among populations, a more marked phenotypic difference would also be expected (Galloway & Fenster, 2000; Pickup et al., 2012). However, our study revealed no or weak relationships between geographic distance and local adaptation. Furthermore, there was no evidence for relationships between quantitative genetic components (P_ST_, total or for single traits) with the environmental components (i.e., environmental distances: LAND, LOC and ENV) of populations, confirming a scarcity of evidence for local adaptation. In any case, though P_ST_ is can be used as an analogue of Q_ST_, results derived from such an index must be considered as a first line of investigation and interpreted with caution since it can be influenced by environmental variability (abiotic conditions) that occurs across sites of sampled populations (Brommer, 2011; Castillo et al., 2015).

It is therorised that, the adaptive ability of a certain species can be the result of either phenotypic plasticity or intraspecific genetic differentiation in response to local environment (Bradshaw, 2006). In the studied populations, the genetic differentiation observed could be attributed to habitat fragmentation and environmental isolation in historical times, which have probably been the major obstacles to gene flow and subsequent genetic drift (Wu et al., 2015). Therefore, due to the intrinsic ability of individuals (which in some species are not able to move from their habitat; see Noel, Machon & Porcher, 2007) to respond plastically to environmental variation (Kostrakiewicz, 2009), the different performance of the studied populations of *P. cornubiense* in response to the different environmental conditions would mainly be due to phenotypic plasticity. In any case, as different selective forces can concomitantly act on natural populations, exactly how species are able to adapt or differentiate across different environments remains a debated question (Olson-Manning, Wagner & Mitchell-Olds, 2012).

### Implications for restoration

The adaptation of populations to local environmental factors can provide key information to conservation practitioners in restoration projects (McKay et al., 2005), such as information on the appropriateness of plant material (seeds or plants) of non-local origin, translocation measures, reinforcement of populations and habitat restoration. The risk of a potential outbreeding depression as a result of combining plant material from divergent populations should be avoided by using populations with analogous genetic patterns and suitable ecological characteristics including both vegetative and reproductive traits (Ellstrand & Elam, 1993; Tallmon, Luikart & Waples, 2004). In our study, as genetic differentiation among fragmented populations of *P. cornubiense* was marked while evidence for local adaptation was not identified across the measured traits, plant translocation actions should particularly take plant geographic provenance, according to identified genetic clusters, into account, as well as the population size of the source population. For a population at high risk, such as the SC population (with low population size, low genetic diversity and no seed production), urgent measures should be applied, carrying out plant translocation from locally adapted seed source populations in order to increase their population size as well as their genetic diversity. The candidate populations to be used in the rescuing of SC are CUS and GRAF populations that belong to a very close cluster of SC. However, the use of GRAF as source population should be limited due to its modest population size. On the other hand, the small population MCB could be restored with individuals coming from the close large population of MCA.

Translocation of propagules (both via seed or from vegetative reproduction) from existing (*in situ*) populations may allow outcrossing, preserving genetic diversity and favouring population size (Gentili et al., 2015). In the studied species, replicating the within-population dynamics of model populations in terms of density, genetic diversity and an adequate ratio between adults (flowering/not flowering) and seedlings/juveniles, may increase the translocation success.

In our study, the analysis of soil factors highlighted a positive relationship between population fitness and levels of Mg. Therefore, specific ex situ and *in situ* trials aiming at investigating the influence of Mg content on the species’ fitness are suggested.

## Conclusions

In this work we try to link genetic differentiation, local adaptation and phenotypic plasticity in populations of a herb forest species needing conservation action. Particularly, the extensive forest fragmentation due to habitat loss that occurred in historical times across the biogeographic province of the Po plain that had an effect on the genetic and demographic structure of the forest herb species *P. cornubiense,* causing a genetic drift in some populations. No phenotypic differentiation due to local adaptation was ascertained, so the variation of phenotypic traits across sites was attributed to plastic response of local populations. Although there are at present no signs of imminent extinction of this plant species across the Po plain, there is evidence that habitat loss and fragmentation have induced a drop in fitness and probably in the migration ability of individuals between forest patches. These factors may lead to future extinction events at local or regional scales if restoration action does not occur. Future restoration action must offset the continuous genetic erosion that this interesting forest herb species suffers from, by translocating suitable plant material into the at-risk populations.

##  Supplemental Information

10.7717/peerj.4929/supp-1 Table S1 Landscape (A) and local variables (B); species traits and demographic data (C); fragmentation table (D); correlation between soil and landscape variables (E)

10.7717/peerj.4929/supp-2 Table S2 Primer combinations

10.7717/peerj.4929/supp-3 Table S3 Multiple test with Holm’s correction values

10.7717/peerj.4929/supp-4 Table S4 Matrixes for Fst, Pst LAND, LOC indexes and variables at the population level

10.7717/peerj.4929/supp-5 Data S1 Phenotypic traits of populations and soil data

10.7717/peerj.4929/supp-6 Data S2 Results of genetic analyses—ISSR marker

10.7717/peerj.4929/supp-7 File S1 STRUCTURE analyses and Neighbour-joining unrooted tree

## Additional Information and Declarations

### Data Availability

The following information was supplied regarding data availability: The raw data are provided in the Supplemental Files.
