# Supplementary material for: Genetic differentiation, local adaptation and phenotypic plasticity in fragmented populations of a rare forest herb
Source: PeerJ. 2018 Jun 13;6:e4929. doi: 10.7717/peerj.4929 (PMC6004105; doi:10.7717/peerj.4929)
Supplement: Table S1 [file peerj-06-4929-s001.pdf]

Supplementary Table ST1: Landscape (a) and local variables (b); species traits and demografic data (c); framentation table (d); correlation between soil and landscape variables (e)

a)

LANDSCAPE VARIABLES		GRAF	SC	RIV1	RIV2	FAGA	FAGB	MCA	MCB	CUS	TO
**Locality**		**Graffignana**	**San Colombano**	**Riv. Bosco Nord**	**Riv. Bosco Sud-Ovest**	**Fagiana A**	**Fagiana B**	**M.te Canto (A)**	**M.te Zocca-Brocchiore (B)**	**Cusago**	**Collina di Superga**
**Municipality**		Graffignana	S.Colombano	Rivanazzano	Rivanazzano	Fagiana	Fagiana	Mapello	Palazzago	Cusago	Torino
**Coord. X (WGS-84; UTM zore 32N)**		534991 E	534991 E	503737 E	503706 E	486526 E	486474 E	541676 E	541387 E	500502 E	401436 E
**Coord. Y (WGS-84; UTM zore 32N)**		5004624 N	5004624 N	4974641 N	4974114 N	5031501 N	5031035 N	5062857 N	5065739 N	5032874 N	4992995 N
		**GRAF**	**SC**	**RIV1**	**RIV2**	**FAGA**	**FAGB**	**MCA**	**MCB**	**CUS**	**TO**
**Habitat size (ha)**	Hab_sz	4	1	2	4	3	2	7.5	5	12	4
**Habitat qualtity (1 = low; 2 = medium; 3 = high)**	Hab_qu	1	1	3	3	2	2	3	2	2	1
**Habitat type (1 = Oak; 2 = Chestnut)**	Hab_ty	2	1	1	1	1	1	2	1	1	2
**Habitat fragmentation**	Hab_fra	2	3	1	1	1	1	1	1	2	1

b)

LOCAL VARIABLES		GRAF	SC	RIV1	RIV2	FAGA	FAGB	MCA	MCB	CUS	TO
**Mg (mg/100g soil)**	Mg	8.73	7.01	16.15	30.19	4.32	3.10	14.38	15.84	5.62	43.58
**Ca (mg/100g soil)**	Ca	17.28	23.86	84.68	69.64	18.33	11.11	92.76	130.10	23.91	24.95
**K (mg/100g soil)**	K	5.42	6.08	10.89	10.37	7.11	6.31	14.02	18.77	11.34	6.12
**pH**	pH	3.6	3.5	4.2	4.1	3.7	3.6	3.9	4.2	3.4	4.1
**Total N (%)**	N%	0.09	0.16	0.16	0.11	0.34	0.50	0.23	0.28	0.40	0.19
**Total C (%)**	C%	0.98	2.29	1.77	1.86	4.89	7.62	2.59	3.49	6.16	2.63
**C/N ratio**	C/N	11.09	14.37	12.06	16.86	14.16	15.07	11.13	12.07	15.42	14.05
**P (mg/100g soil)**	P	0.01	0.12	0.09	0.09	0.39	0.37	0.17	0.38	1.37	0.20
**Slope**	Slope	30.00	15.00	20.00	25.00	0.00	0.00	27.00	20.00	0.00	30.00
**Alien species presence**	Alien	1	1	0	0	1	1	0	0	1	1
Metcalfa pruinosa presence	Metca	1	1	1	0	1	1	0	0	1	1
**Elevation**	Elev	130	16	235	370	126	111	330	470	140	300

c)

DEMOGRAFIC DATA AND TRAITS	Abbreviation	GRAF	SC	RIV1	RIV2	FAGA	FAGB	MCA	MCB	CUS	TO
**Flowering adult**	FLad	22	3	13	94	90	8	64	22	9	420
**Non-floweing adult**	Non_FLad	15	10	6	182	73	31	163	34	146	190
**Seedling/Juvenile**	Seedl/Juv	13	3	13	102	73	28	173	28	54	45
**Pop size**	Pop_sz	50	16	32	378	236	67	400	84	209	655
**Inflorescence size**	Infl_sz	6.6	6.2	7.0	7.9	5.9	5.5	5.8	5.3	5.0	8.4
**N° comp umb**	Comp_umb	2.7	2.3	4.8	3.3	3.9	2.3	4.5	1.3	2.8	4.0
**N° simp umb**	Simp_umb	14.4	17.3	16.8	14.9	15.0	14.9	13.5	12.8	15.9	17.0
**Plant height**	Pl_H	77.9	92.7	99.2	82.5	85.2	90.2	71.0	42.2	68.9	94.7
**Lateral spread**	Lat_spr	47.8	47.1	48	41.6	34.3	42.3	45.0	33.3	54.4	49.2
**N° leaves**	N_leav	2.4	3.0	3.2	2.1	2.5	3.3	2.5	2.7	3.3	3.1
**Seed weight**	SW	0.0157	0.0	0.0163	0.0177	0.0158	0.0171	0.0349	0.0089	0.0045	0.0105
**Genetic diversity**	H_Nei	0.130	0.087	0.114	0.119	0.099	0.121	0.084	0.071	0.155	0.112

d)

FRAGMENTATION TABLE	**SCORING: 1 = 1-10; 2 = 11-20; 3 = 20-30**									
	GRAF	SC	RIV1	RIV2	FAGA	FAGB	MCA	MCB	CUS	TO
**Distance from the closest patch (1-10):** 1= less than 0.5 km; 2= 0.5-1 km; 3= 1-2 km; 4=2-5 km; 5= 5-10 km; 6= 10-20 km; 7=-20-40 km; 8=40-80 km; 9=80-100 km; 10 = more than 100 km	3	3	1	1	2	2	4	4	7	9
**Dissection/perforation (0-10):** presence of forest roads, paths, buildings, clearings, etc	6	10	1	1	5	4	2	2	4	0
**Shrinkage (1-10):** 1 very small and linear-10 big and rounded	5	9	4	1	1	3	1	4	2	1
	**14**	**22**	**6**	**3**	**8**	**9**	**7**	**10**	**13**	**10**

e)

CORRELATION BETWEEN SOIL/ENVIRONMENTAL VARIABLES ACCORDING TO SPEARMAN

	Mg	Ca	K	pH	N%	C%	C/N	P	Hab_sz	Hab_qu	Hab_ty	Hab_fra
**Mg**		0.028883	0.67607	0.01333	0.053718	0.089724	0.5334	0.1869	0.58551	0.61918	0.33359	0.34083
**Ca**	0.68485		0.009222	0.015801	0.65148	0.55631	0.51089	0.93377	0.18593	0.10762	0.91702	0.24625
**K**	0.15152	0.7697		0.20042	0.29305	0.42504	0.98674	0.22911	0.072077	0.019077	0.45774	0.1925
**pH**	0.74545	0.73333	0.44242		0.34659	0.34659	0.34659	0.48878	0.79977	0.1925	0.91702	0.019077
**N%**	-0.62424	-0.16364	0.3697	-0.33333		1.47E-06	0.31038	0.00034361	0.63403	0.90188	0.33359	0.57522
**C%**	-0.56364	-0.21212	0.28485	-0.33333	0.97576		0.16152	0.00011203	0.62177	0.90188	0.33359	0.57522
**C/N**	-0.22424	-0.23636	0.0060606	-0.33333	0.35758	0.47879		0.22911	0.78667	0.80494	0.043703	0.85319
**P**	-0.45455	-0.030303	0.41818	-0.24848	0.90303	0.92727	0.41818		0.31126	0.90188	0.33359	0.71034
**Hab_sz**	0.19695	0.45544	0.59084	0.092319	0.17233	0.17848	-0.098473	0.35697		0.56918	0.32565	0.85093
**Hab_qu**	0.17979	0.53936	0.71915	0.44947	0.044947	-0.044947	0.089893	-0.044947	0.2054		0.43037	0.076698
**Hab_ty**	0.34188	0.037987	-0.26591	0.037987	-0.34188	-0.34188	-0.64578	-0.34188	0.34718	-0.28172		1
**Hab_fra**	-0.3371	-0.40452	-0.44947	-0.71915	-0.20226	-0.20226	0.06742	-0.13484	-0.068465	-0.58333	8.40E-20	
