# Supplementary material for: Genetic differentiation, local adaptation and phenotypic plasticity in fragmented populations of a rare forest herb
Source: PeerJ. 2018 Jun 13;6:e4929. doi: 10.7717/peerj.4929 (PMC6004105; doi:10.7717/peerj.4929)
Supplement: Table S2 [file peerj-06-4929-s002.pdf]

SUPPLEMENTARY TABLE ST2: Primer combinations

Code	Primer ISSR	T annealing °C
UBC807	AGA GAG AGA GAG AGA GT	50
UBC808	AGA GAG AGA GAG AGA GC	52
UBC811	GAG AGA GAG AGA GAG AC	52
UBC825	ACA CAC ACA CAC ACA CT	50
UBC834	AGA GAG AGA GAG AGA GCT	52
UBC841	GAG AGA GAG AGA GAG AYC	54
UBC842	CTC TCT CTC TCT CTC TYG	54
UBC847	CAC ACA CAC ACA CAC ARG	52
