# Supplementary material for: Genetic differentiation, local adaptation and phenotypic plasticity in fragmented populations of a rare forest herb
Source: PeerJ. 2018 Jun 13;6:e4929. doi: 10.7717/peerj.4929 (PMC6004105; doi:10.7717/peerj.4929)
Supplement: Table S3 [file peerj-06-4929-s003.pdf]

SUPPLEMENTARY TABLE ST3: multiple test with Holm's correction values

a) GLM / GLMM tests

Variabiles	Family	Model	p vlue	Holm correction
FLad vs Hab_fra	A	GLM	0.32478	1
FLad vs Hab_ty	A	GLM	0.08916	1
FLad vs Hab_qu	A	GLM	0.38151	1
FLad vs Hab_sz	A	GLM	0.90242	1
FLad vs Elev	A	GLM	0.40165	1
FLad vs Metca	A	GLM	0.82193	1
FLad vs Alien	A	GLM	0.60664	1
FLad vs Slope	A	GLM	0.21728	1
FLad vs P	A	GLM	0.63545	1
FLad vs C/N	A	GLM	0.71051	1
FLad vs C%	A	GLM	0.61531	1
FLad vs N%	A	GLM	0.44776	1
FLad vs pH	A	GLM	0.22424	1
FLad vs K	A	GLM	0.45313	1
FLad vs Ca	A	GLM	0.63983	1
**FLad vs Mg**	A	GLM	**0.00019**	**0.015**
Non_FLad vs Hab_fra	A	GLM	0.32229	1
Non_FLad vs Hab_ty	A	GLM	0.30685	1
Non_FLad vs Hab_qu	A	GLM	0.48066	1
Non_FLad vs Hab_sz	A	GLM	0.06428	1
Non_FLad vs Elev	A	GLM	0.18556	1
Non_FLad vs Metca	A	GLM	0.25572	1
Non_FLad vs Alien	A	GLM	0.71817	1
Non_FLad vs Slope	A	GLM	0.52968	1
Non_FLad vs P	A	GLM	0.47926	1
Non_FLad vs C/N	A	GLM	0.24254	1
Non_FLad vs C%	A	GLM	0.99076	1
Non_FLad vs N%	A	GLM	0.91778	1
Non_FLad vs pH	A	GLM	0.52564	1
Non_FLad vs K	A	GLM	0.73396	1
Non_FLad vs Ca	A	GLM	0.92680	1
Non_FLad vs Mg	A	GLM	0.024368	1
Seedl/Juv vs Hab_fra	A	GLM	0.18007	1
Seedl/Juv vs hab_ty	A	GLM	0.34511	1
Seedl/Juv vs Hab_qu	A	GLM	0.036014	1
Seedl/Juv vs Hab_sz	A	GLM	0.16499	1
Seedl/Juv vs Elev	A	GLM	0.19339	1
Seedl/Juv vs Metca	A	GLM	0.019779	1
Seedl/Juv vs Alien	A	GLM	0.18032	1
Seedl/Juv vs Slope	A	GLM	0.64179	1
Seedl/Juv vs P	A	GLM	0.96323	1
Seedl/Juv vs C/N	A	GLM	0.99432	1
Seedl/Juv vs C%	A	GLM	0.94512	1
Seedl/Juv vs N%	A	GLM	0.97536	1
Seedl/Juv vs pH	A	GLM	0.59807	1
Seedl/Juv vs K	A	GLM	0.30104	1
Seedl/Juv vs Ca	A	GLM	0.37026	1
Seedl/Juv vs Mg	A	GLM	0.59937	1
Pop_sz vs vs Hab_fra	A	GLM	0.18411	1
Pop_sz vs vs Hab_ty	A	GLM	0.090819	1
Pop_sz vs Hab_qu	A	GLM	0.86976	1
Pop_sz vs Hab_sz	A	GLM	0.40358	1
Pop_sz vs Elev	A	GLM	0.17813	1
Pop_sz vs Metca	A	GLM	0.47268	1
Pop_sz vs Alien	A	GLM	0.89993	1
Pop_sz vs Slope	A	GLM	0.27431	1
Pop_sz vs P	A	GLM	0.98443	1
Pop_sz vs C/N	A	GLM	0.52875	1
Pop_sz vs C%	A	GLM	0.75433	1
Pop_sz vs N%	A	GLM	0.71420	1
Pop_sz vs pH	A	GLM	0.26990	1
Pop_sz vs K	A	GLM	0.94178	1
Pop_sz vs Ca	A	GLM	0.97367	1
**Pop_sz vs Mg**	A	GLM	**0.00051**	**0.04029**

b) MANTEL TEST

Variabiles	Family	p value	Holm correction
Fst vs SW	A	0.4133	1
Fst vs Simp_umb	A	0.4842	1
Fst vs Pl_H	A	0.3194	1
Fst vs N_leav	A	0.2487	1
Fst vs Lat_spr	A	0.4153	1
Fst vs Inlf_sz	A	0.3814	1
Fst vs Pstm	A	0.3129	1
Geo vs SW	B	0.3089	1
Geo vs Simp_umb	B	0.3911	1
Geo vs Pl_H	B	0.0859	1
Geo vs N_leav	B	0.1814	1
Geo vs Lat_spr	B	0.0652	1
Geo vs Inlf_sz	B	0.2171	1
Geo vs Pstm	B	0.0195	0.9165
Geo vs Fst	B	0.4232	1
LOC_m vs SW	B	0.4599	1
LOC_m vs Simp_umb	B	0.1445	1
LOC_m vs Pl_H	B	0.1358	1
LOC_m vs N_leav	B	0.2193	1
LOC_m vs Lat_spr	B	0.0355	1
LOC_m vs Inlf_sz	B	0.1238	1
LOC_m vs Pstm	B	0.0717	1
LOC_m vs Fst	B	0.2106	1
LOC_m vs Geo	B	0.0931	1
LAND_m vs SW	B	0.0497	1
LAND_m vs Simp_umb	B	0.2401	1
LAND_m vs Pl_H	B	0.2559	1
LAND_m vs N_leav	B	0.3788	1
LAND_m vs Lat_spr	B	0.1414	1
LAND_m vs Inlf_sz	B	0.0380	1
LAND_m vs Pstm	B	0.3194	1
LAND_m vs Fst	B	0.0331	1
LAND_m vs Geo	B	0.4546	1
LAND_m vs LOC_m	B	0.3756	1
ENV_ok vs SW	B	0.3236	1
ENV_ok vs Simp_umb	B	0.1023	1
ENV_ok vs Pl_H	B	0.1709	1
ENV_ok vs N_leav	B	0.1691	1
ENV_ok vs Lat_spr	B	0.0439	1
ENV_ok vs Inlf_sz	B	0.0425	1
ENV_ok vs Pstm	B	0.0788	1
ENV_ok vs Fst	B	0.1881	1
ENV_ok vs Geo	B	0.1267	1
**ENV_ok vs LOC_m**	B	**0.0001**	**0.005**
ENV_ok vs LAND_m	B	0.0427	1
LOC vs Comp_umb	B	0.3470	1
LAND vs Comp_umb	B	0.4382	1
GEO vs Comp_umb	B	0.0118	0.5664
ENV vs Comp_umb	B	0.3058	1

H_Nei vs Hab_fra	A	GLM	0.68307	1
H_Nei vs Hab_ty	A	GLM	0.96637	1
H_Nei vs Hab_qu	A	GLM	0.8513	1
H_Nei vs Hab_sz	A	GLM	0.22185	1
H_Nei vs Elev	A	GLM	0.248411	1
H_Nei vs Metca	A	GLM	0.10311	1
H_Nei vs Alien	A	GLM	0.1839	1
H_Nei vs Slope	A	GLM	0.43934	1
H_Nei vs P	A	GLM	0.10197	1
H_Nei vs C/N	A	GLM	0.22143	1
H_Nei vs C%	A	GLM	0.43111	1
H_Nei vs N%	A	GLM	0.63037	1
H_Nei vs pH	A	GLM	0.17416	1
H_Nei vs K	A	GLM	0.019249	1
H_Nei vs Ca	A	GLM	0.047321	1
H_Nei vs Mg	A	GLM	0.84611	1
Comp_umb vs Alien	B	GLMM	0.3714	1
Comp_umb vs C%	B	GLMM	0.3708	1
Comp_umb vs C/N	B	GLMM	0.7046	1
Comp_umb vs Ca	B	GLMM	0.9492	1
Comp_umb vs Elev	B	GLMM	0.831	1
**Comp_umb vs Hab_fra**	**B**	GLMM	0.3231	1
Comp_umb vs Hab_qu	B	GLMM	0.5179	1
Comp_umb vs Hab_sz	B	GLMM	0.9369	1
Comp_umb vs Hab_ty	B	GLMM	0.7776	1
Comp_umb vs K	B	GLMM	0.5889	1
Comp_umb vs Metca	B	GLMM	0.741	1
Comp_umb vs Mg	B	GLMM	0.3862	1
Comp_umb vs N%	B	GLMM	0.389	1
Comp_umb vs P	B	GLMM	0.4893	1
Comp_umb vs pH	B	GLMM	0.4576	1
Comp_umb vs Slope	B	GLMM	0.519	1
Infl_sz vs Alien	B	GLMM	0.7681	1
Infl_sz vs C%	B	GLMM	0.0698	1
Infl_sz vs C/N	B	GLMM	0.5753	1
Infl_sz vs Ca	B	GLMM	0.7941	1
Infl_sz vs Elev	B	GLMM	0.5394	1
Infl_sz vs Hab_fra	B	GLMM	0.5413	1
Infl_sz vs Hab_qu	B	GLMM	0.3366	1
Infl_sz vs Hab_sz	B	GLMM	0.2429	1
Infl_sz vs Hab_ty	B	GLMM	0.6056	1
Infl_sz vs K	B	GLMM	0.2593	1
Infl_sz vs Metca	B	GLMM	0.9281	0.4568
Infl_sz vs Mg	B	GLMM	0.0032	1
Infl_sz vs N%	B	GLMM	0.0313	1
Infl_sz vs P	B	GLMM	0.0852	1
Infl_sz vs pH	B	GLMM	0.1218	1
Infl_sz vs Slope	B	GLMM	0.0607	1
Lat_spr vs Alien	B	GLMM	0.6297	1
Lat_spr vs C%	B	GLMM	0.7142	1
Lat_spr vs C/N	B	GLMM	0.9226	1
Lat_spr vs Ca	B	GLMM	0.2704	1
Lat_spr vs Elev	B	GLMM	0.2885	1
Lat_spr vs Hab_fra	B	GLMM	0.1875	1
Lat_spr vs Hab_qu	B	GLMM	0.4425	1
Lat_spr vs Hab_sz	B	GLMM	0.3289	1
Lat_spr vs Hab_ty	B	GLMM	0.6369	1
Lat_spr vs K	B	GLMM	0.3112	1
Lat_spr vs Metca	B	GLMM	0.1891	1
Lat_spr vs Mg	B	GLMM	0.7371	1
Lat_spr vs N%	B	GLMM	0.5859	1
Lat_spr vs P	B	GLMM	0.4839	1
Lat_spr vs pH	B	GLMM	0.3668	1
Lat_spr vs Slope	B	GLMM	0.6611	1
N_leav vs Alien	B	GLMM	0.4259	1
N_leav vs C%	B	GLMM	0.1914	1
N_leav vs C/N	B	GLMM	0.7952	1

N_leav vs Ca	B	GLMM	0.5022	1
N_leav vs Elev	B	GLMM	0.3543	1
N_leav vs Hab_fra	B	GLMM	0.6665	1
N_leav vs Hab_qu	B	GLMM	0.4288	1
N_leav vs Hab_sz	B	GLMM	0.9309	1
N_leav vs Hab_ty	B	GLMM	0.8581	1
N_leav vs K	B	GLMM	0.6923	1
N_leav vs Metca	B	GLMM	0.0662	1
N_leav vs Mg	B	GLMM	0.8203	1
N_leav vs N%	B	GLMM	0.2008	1
N_leav vs P	B	GLMM	0.1977	1
N_leav vs pH	B	GLMM	0.616	1
N_leav vs Slope	B	GLMM	0.2544	1
Pl_H vs Hab_fra	B	GLMM	0.6153	1
Pl_H vs Hab_ty	B	GLMM	0.7999	1
Pl_H vs Alien	B	GLMM	0.4684	1
Pl_H vs C%	B	GLMM	0.0851	1
Pl_H vs C/N	B	GLMM	0.121	1
Pl_H vs Ca	B	GLMM	0.862	1
Pl_H vs Elev	B	GLMM	0.4616	1
Pl_H vs Hab_qu	B	GLMM	0.1201	1
Pl_H vs Hab_sz	B	GLMM	0.8205	1
Pl_H vs K	B	GLMM	0.0086	1
Pl_H vs Metca	B	GLMM	0.0573	1
Pl_H vs Mg	B	GLMM	0.645	1
Pl_H vs N%	B	GLMM	0.6351	1
Pl_H vs P	B	GLMM	0.3524	1
Pl_H vs pH	B	GLMM	0.7957	1
Pl_H vs Slope	B	GLMM	0.9365	1
Simp_umb vs Alien	B	GLMM	0.3518	1
Simp_umb vs C%	B	GLMM	0.8831	1
Simp_umb vs C/N	B	GLMM	0.3088	1
Simp_umb vs Ca	B	GLMM	0.1321	1
Simp_umb vs Elev	B	GLMM	0.2309	1
Simp_umb vs Hab_fra	B	GLMM	0.3592	1
Simp_umb vs Hab_qu	B	GLMM	0.3229	1
Simp_umb vs Hab_sz	B	GLMM	0.5024	1
Simp_umb vs Hab_ty	B	GLMM	0.9943	1
Simp_umb vs K	B	GLMM	0.0714	1
Simp_umb vs Metca	B	GLMM	0.0275	1
Simp_umb vs Mg	B	GLMM	0.4652	1
Simp_umb vs N%	B	GLMM	0.6836	1
Simp_umb vs P	B	GLMM	0.888	1
Simp_umb vs pH	B	GLMM	0.7408	1
Simp_umb vs Slope	B	GLMM	0.794	1
SW vs Alien	B	GLMM	0.3984	1
SW vs C%	B	GLMM	0.5048	1
SW vs C/N	B	GLMM	0.3032	1
SW vs Ca	B	GLMM	0.4325	1
SW vs Elev	B	GLMM	0.701	1
**SW vs Hab_fra**	**B**	GLMM	0.3375	1
SW vs Hab_qu	B	GLMM	0.4183	1
SW vs Hab_sz	B	GLMM	0.8068	1
SW vs Hab_ty	B	GLMM	0.5697	1
SW vs K	B	GLMM	0.7335	1
SW vs Metca	B	GLMM	0.1898	1
SW vs Mg	B	GLMM	0.8454	1
SW vs N%	B	GLMM	0.6401	1
SW vs P	B	GLMM	0.1582	1
SW vs pH	B	GLMM	0.7364	1
SW vs Slope	B	GLMM	0.4217	1
