# Supplementary material for: Genetic differentiation, local adaptation and phenotypic plasticity in fragmented populations of a rare forest herb
Source: PeerJ. 2018 Jun 13;6:e4929. doi: 10.7717/peerj.4929 (PMC6004105; doi:10.7717/peerj.4929)
Supplement: Table S4 [file peerj-06-4929-s004.pdf]

Supplementary Table ST3. Matrixes for Fst, Pst LAND, LOC indexes and variables at the population level.

1 10

Fst

GRAF	SC	RIV1	RIV2	FagA	FagB	MCA	MCB	CUS	TO	
0.000										GRAF
0.362	0.000									SC
0.158	0.413	0.000								RIV1
0.285	0.492	0.128	0.000							RIV2
0.272	0.496	0.189	0.328	0.000						FagA
0.259	0.483	0.170	0.313	0.110	0.000					FagB
0.293	0.554	0.263	0.414	0.329	0.303	0.000				MCA
0.304	0.574	0.230	0.393	0.312	0.299	0.213	0.000			MCB
0.169	0.498	0.225	0.361	0.346	0.348	0.397	0.413	0.000		CUS
0.162	0.477	0.125	0.209	0.273	0.259	0.318	0.316	0.189	0.000	TO

1 10

Land

GRAF	SC	RIV1	RIV2	FAGA	FAGB	MCA	MCB	CUS	TO	
0										GRAF
2.6833	0									SC
3.5661	3.7787	0								RIV1
3.5116	3.8795	0.62142	0							RIV2
2.8156	3.173	1.2635	1.2635	0						FAGA
2.8666	3.127	1.2247	1.3734	0.31071	0					FAGB
3.0378	4.7483	2.6844	2.3384	2.7822	2.9506	0				MCA
2.8156	3.3506	1.5391	1.2635	0.62142	0.93213	2.5277	0			MCB
3.4589	3.9022	3.6331	3.1183	3.1409	3.4205	3.1283	2.6031	0		CUS
1.4302	3.6519	3.2668	3.2071	2.4253	2.4843	2.68	2.4253	3.743	0	TO

1 10

Soil

GRAF	SC	RIV1	RIV2	FAGA	FAGB	MCA	MCB	CUS	TO	
0										GRAF
2.4104	0									SC
3.7128	4.0503	0								RIV1
5.3452	5.11	3.5933	0							RIV2
4.0569	2.5061	4.4106	5.5428	0						FAGA
5.5021	3.9305	5.7567	6.5593	1.8688	0					FAGB
4.5198	5.0117	2.6639	3.5614	5.134	6.176	0				MCA
6.3321	6.4919	3.6858	4.0818	6.0449	6.893	2.2481	0			MCB
6.034	4.5582	6.089	6.7108	3.001	3.0664	6.2832	6.681	0		CUS
3.9041	4.1788	3.7172	3.7811	4.6111	5.6474	4.696	5.633	6.0921	0	TO

1 10

Env

GRAF	SC	RIV1	RIV2	FAGA	FAGB	MCA	MCB	CUS	TO	
0										GRAF
3.3113	0									SC
4.9454	4.7436	0								RIV1
6.2335	5.7429	3.6467	0							RIV2
4.7266	2.8578	4.588	5.685	0						FAGA
6.0369	4.1286	5.8855	6.7015	1.8945	0					FAGB
5.2547	6.2834	3.7819	4.2605	5.8394	6.8447	0				MCA
6.7807	6.7223	3.9942	4.2729	6.0767	6.9558	3.3828	0			MCB
6.9551	5.8274	6.9448	7.2604	4.1019	4.3654	6.8717	7.0261	0		CUS
3.9041	4.7557	4.9487	4.9581	5.21	6.1697	5.4069	6.133	7.0056	0	TO

1 10

Geo

GRAF	SC	RIV1	RIV2	FagA	FagB	MCA	MCB	CUS	TO	
0.000										GRAF
2422.973	0.000									SC
43310.422	43204.738	0.000								RIV1
43699.102	43572.918	527.911	0.000							RIV2
55418.672	57683.145	59407.727	59903.422	0.000						FagA
55239.844	57497.652	58977.066	59472.195	468.892	0.000					FagB
58615.453	60316.152	96028.281	96524.820	63440.691	63717.348	0.000				MCA
61448.777	63165.320	98571.641	99070.672	64668.152	64960.027	2896.454	0.000			MCB
44581.988	46962.945	58322.789	58847.289	14043.279	14148.028	50934.063	52456.566	0.000		CUS
134060.328	135455.438	103934.422	103998.297	93397.109	93158.484	156677.875	157727.531	106791.422	0.000	TO

1 10

Psttotal

GRAF	SC	RIV1	RIV2	FAGA	FAGB	MCA	MCB	CUS	TO	
0										GRAF
0.10341	0									SC
0.0179	0.05353	0								RIV1
0.11024	0.05527	0.10063	0							RIV2
0.09608	0.23301	0.17657	0.36332	0						FAGA
0.41247	0.01003	0.16341	0.26317	0.02171	0					FAGB
0.08012	0.08368	0.14719	0.21033	0.23976	0.24788	0				MCA
0.1276	0.07573	0.08035	0.36845	0.1997	0.13879	0.10127	0			MCB
0.37922	0.14494	0.16724	0.05321	0.10971	0.35377	0.15352	0.26983	0		CUS
0.05797	0.40254	0.08953	0.2253	0.2467	0.06295	0.11028	0.4226	0.15598	0	TO

Mantel Results for Fst vs Geo

**X Matrix**	Fst
**Y Matrix**	Geo
**Data Title**	Fst

**No. Samples**	10	**No. in Matrix**	45
**No. Permutations**	9999		

**SSx**	**SSy**	**SPxy**	**Rxy**	**P(rxy-rand >= rxy-data)**
0.611	62154903790.9	2595.893	0.013	0.415
