# Supplementary material for: Genetic differentiation, local adaptation and phenotypic plasticity in fragmented populations of a rare forest herb
Source: PeerJ. 2018 Jun 13;6:e4929. doi: 10.7717/peerj.4929 (PMC6004105; doi:10.7717/peerj.4929)
Supplement: File S1 [file peerj-06-4929-s007.docx]

**SUPPLEMENTARY FILE 1: STRUCTURE analyses and Neighbour-joining unrooted tree**

1. Results of the ΔK calculation performed with STRUCTURE without using the LOCPRIOR option.


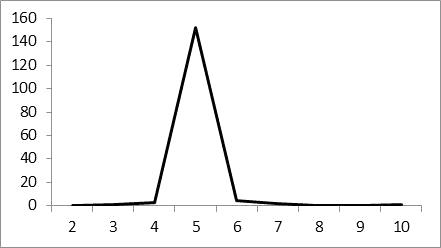


B) Neighbour-joining unrooted tree based on ISSR markers (Nei and Li distances). Bootstrap values were >40% for all nodes.
